# Improving maternal and newborn care: cost-effectiveness of an innovation to rebrand traditional birth attendants in Sierra Leone

**DOI:** 10.1007/s00038-020-01487-z

**Published:** 2020-10-10

**Authors:** Jean Christophe Fotso, Ashley Ambrose, Paul Hutchinson, Disha Ali

**Affiliations:** 1 EVIHDAF (Evidence for Sustainable Human Development Systems in Africa), BP 35328, Yaoundé, Cameroon; 2 CPC Community Health, Denver, CO USA; 3 School of Public Health and Tropical Medicine, Tulane University New Orleans, New Orleans, LA USA; 4 JSI Research and Training Institute, Inc., Rosslyn, VA USA

**Keywords:** Maternal and newborn, Innovations, Traditional birth attendant, Social enterprise, Cost-effectiveness, Sierra Leone

## Abstract

**Objectives:**

This paper evaluates the cost-effectiveness of rebranding former traditional birth attendants (TBAs) to conduct health promotion activities and refer women to health facilities.

**Methods:**

The project used 200 former TBAs, 100 of whom were also enrolled in a small income generating business. The evaluation had a three-arm, quasiexperimental design with baseline and endline household surveys. The three arms were: (a) Health promotion (HP) only; (b) Health promotion plus business (HP+); and (c) the comparison group. The Lives Saved Tool is used to estimate the number of lives saved.

**Results:**

The HP+ intervention had a statistically significant impact on health facility delivery and four or more antenatal care (ANC) visits during pregnancy. The cost-effectiveness ratio was estimated at US$4130 per life year saved in the HP only arm, and US$1539 in the HP+ arm. Therefore, only the HP+ intervention is considered to be cost-effective.

**Conclusions:**

It is critical to prioritize cost-effective interventions such as, in the case of rural Sierra Leone, community-based strategies involving rebranding TBAs as health promoters and enrolling them in health-related income generating activities.

## Introduction

In Sierra Leone, where about 40% of births are conducted without a skilled health provider, maternal mortality is estimated to be highest in the world, at 1360 deaths per 100,000 live births (WHO 2015a, b). Neonatal mortality also remains among the highest in the world at 33.2 neonatal deaths per 1000 live births (UN IGME 2017; Statistics Sierra Leone and ICF International 2014). Of the births unattended by a skilled health professional, over 90% are assisted by a traditional birth attendant (TBA). Yet reductions in both maternal and newborn mortality require access to skilled attendance during labor and delivery (WHO 2015a, b).

In many rural settings, TBAs are relied on by mothers and families, as they provide accessible and affordable care to mothers who may otherwise have no access to health services (Dorwie and Pacquiao 2014). In 2010, as a strategy to increase the utilization of facility services for maternal and newborn health care, the Government of Sierra Leone introduced free health care for pregnant women, lactating mothers and children under the age of 5 years (Government of Sierra Leone 2009). Yet despite these efforts, women continue to seek advice and services from TBAs outside of health facilities (Shiferaw et al. 2013; Treacy and Sagbakken 2015).

Reasons for not using facility care include long distances to health facilities and an entrenched culture in rural Sierra Leone which regards normal and safe delivery as one that takes place in the village, with the hospital predominantly seen as a place to go only if complications occur or are likely to occur. The prohibitive cost of services and the geographic inaccessibility of health facilities, coupled with poor quality and disrespectful care, are also cited among the main barriers to utilization of formal maternal health services in Sierra Leone (Treacy and Sagbakken 2015; Oyerinde et al. 2013). These findings have also been reported in other countries like Ethiopia, Malawi and Nigeria (Shiferaw et al. 2013; Olufunke and Akintujoye 2012; WHO 2010; Garces et al. 2012).

### Health promotion roles for TBAs

Given TBAs’ standing roles in their communities as respected and well-utilized sources of care, the WHO suggests reshaping the role of TBAs to link communities with health facilities to contribute to a greater continuum of care (WHO 2010). Although largely illiterate and with little formal education, TBAs are well positioned to reach mothers and newborns with targeted health information and services, and link them with the formal health care system (Garces et al. 2012; WHO 2015a, b). As countries strive to promote skilled birth attendance and recommendations on ways in which TBAs should be included in the formal health sector evolve (WHO 2015a, b), the role of TBAs needs to be redefined in resource-poor settings (Kayombo 2013; Campbell and Graham 2006).

Evidence suggests that training TBAs in non-delivery roles has the potential to result in promising improvements in facility attendance and maternal and newborn health outcomes (Campbell and Graham 2006). TBAs have demonstrated competence in the promotion of antenatal care (ANC), health facility delivery, postnatal care (PNC) and the awareness of danger signs for mothers and infants (Sibley et al. 2004; Matendo et al. 2011). However, few attempts have been made to analyze the cost-effectiveness of TBA training programs, making national scale-up plans difficult (Wilson et al. 2011).

### Cost-effectiveness of community-based MNCH interventions

Maternal, newborn and child health (MNCH) evaluation studies are increasing including cost-effectiveness analyses to produce evidence not only on what works, but also on the strategies with the highest value for money (Maitra et al. 2016; Adam et al. 2005). Several studies have reported that strategies involving community health workers (CHWs) and TBAs can be cost-effective (Curry et al. 2013; Sabin et al. 2012). However, little is known about cost-effectiveness of health system strengthening interventions to improve primary care, particularly strategies that use available community resources like TBAs. A perennial challenge in community-based programming is the motivation of CHWs to remain in their positions once trained, and to fulfill their responsibilities effectively over time. While the concept of motivation remains complex and multifaceted, involving a host of psychological, interpersonal and contextual factors, direct financial incentives have been shown to be associated with improved engagement and effectiveness (Singh et al. 2015).

This paper evaluates the effectiveness and assesses the cost-effectiveness of a behavior change intervention delivered by former TBAs trained and rebranded as health promoters. In their new roles, these health promoters conduct health promotion activities in their communities and refer women to health facilities for maternal and newborn care. This paper also tests the extent to which incentivizing TBAs results in added effectiveness and cost-effectiveness of programs that collaborate with TBAs for improved maternal and newborn health.

## Methods

### The intervention

The Essential Newborn Care Corps (ENCC) intervention was aimed at increasing facility attendance for delivery, antenatal and postnatal care services through the recruitment, training and rebranding of TBAs to work as Maternal Newborn Health Promoters (MNHPs). In their new roles, MNHPs provide health counseling and referrals to pregnant women and new mothers. ENCC was implemented in Bo District of Sierra Leone between March 2014 and September 2016. The intervention covers the catchment areas of 18 primary health facilities, known as peripheral health units (PHUs), with a total population of about 105,000.

At the beginning of the project, 200 TBAs received a 2-week training on the promotion of birth preparedness, complication readiness and newborn care. Newly branded as MNHPs with ID cards, shirts and pictorial counseling cards, they conducted home visits. During these home visits, they gave health promotion messages, checked for maternal and newborn complications and provided referrals to health facilities for ANC, PNC, delivery, maternal and newborn complications and family planning. Additional project components included monthly meetings and supportive supervision for MNHPs, facilitated by project staff and health facility staff, as well as quarterly review meetings with project stakeholders.

The project also sought to explore how providing MNHPs the opportunity to sell health and baby products as a source of income might incentivize them in their new role and further contribute to positive health outcomes. Half of the MNHPs were enrolled in a small social enterprise. They received a 5-day business training and were provided with a no-interest loan in the form of a product basket valuing approximately US$30. They sold health and baby products to women during their home visits, made loan repayments and purchased products to build their businesses during monthly meetings. The products were sourced from wholesalers in Sierra Leone’s capital city and sold to MNHPs at a 7% profit margin. The convenience of bringing products to the doorsteps of women living in rural areas, and the affordable pricing of the products was to contribute to the viability of the business model.

### Data

#### Effectiveness of the intervention

To assess the impact of the intervention on target MNCH outcomes and the extent to which the business model added value, the evaluation used a three-arm, quasi-experimental design that isolated as best as possible the effects of the intervention from broader secular or contextual changes. The three arms were defined as follows:

- Health promotion (HP) only arm: 100 rebranded TBAs in the catchment areas of nine PHUs with a population of about 57,040.
- Health promotion plus business (HP+) arm: 100 rebranded TBAs in the catchment areas of nine PHUs with a population estimated at 46,355.
- Comparison arm: Catchment areas of nine PHUs with a population of nearly 54,700.

Baseline and endline household surveys were conducted with women age 15–49 who had a live birth in the year prior to the survey, or who were pregnant at the time of the survey. The surveys were administered in October–December 2013 and June–July 2016. In each survey, a two-stage sampling procedure was utilized, with enumeration areas (EAs) selected in the first stage using probability proportional to size (PPS). In the second stage, a complete listing of households was carried out in each selected EA, after which a number of households were systematically selected using a sampling interval determined from the total number of households in the EA and the sample of households needed for each EA. The baseline questionnaires were drawn largely from the 2008 Sierra Leone demographic and health surveys (DHS) and adapted to the context of Bo District. The endline tools were similar to the baseline tools, and included variables on the exposure to the intervention. The final sample included 795 eligible women from 66 EAs at baseline, and 1110 women from 92 EAs at endline.

In this paper, we examine the impact of the intervention on its six primary health outcomes: (1) Initiation of ANC during the first trimester of pregnancy, (2) Four or more ANC visits during pregnancy, (3) Health facility delivery, (4) PNC for mothers by a health professional, (5) PNC for newborns by a health professional and (6) Initiation of breastfeeding within 1 h of delivery. The baseline and endline surveys also collected data on three other MNCH-related outcomes which are part of the variables required to estimate the lives saved: (1) Tetanus toxoid vaccination during pregnancy, (2) Iron supplementation during pregnancy and (3) Preventive treatment of malaria during pregnancy.

This study focuses on women who had a child in the year preceding the surveys. In both the baseline and endline surveys, the majority of the study population had no education and were predominantly Muslim. Just over half of the women were between the ages of 20 and 29 years. The analyses control for the following variables: household wealth and women’s education, age and religion; the distributions of which are shown in Table 1.

**Table 1:** Characteristics of respondents at baseline and endline (Essential Newborn Care Corps Evaluation, Sierra Leone, 2014–2016)

	Baseline	Endline
Household wealth		
Low 40%	37.5	44.0
Middle 20%	22.4	18.1
High 40%	40.2	38.0
Women’s education		
None	64.5	59.8
Primary	15.9	16.7
Secondary+	19.6	23.5
Women’s age		
< 19	13.5	19.9
20–29	54.1	53.5
30+	32.4	26.6
Women’s religion		
Christian	25.7	31.6
Muslim	74.3	68.4
Evaluation arm		
Comparison arm	32.2	40.4
Health Promotion (HP) only arm	32.6	24.6
Health Promotion plus Business (HP+) arm	35.3	35.1
*N*	510	753

Among women who had a live birth in the 2 years preceding the survey

#### Intervention costs

The cost analysis of the ENCC project followed a narrow health sector perspective, covering both the financial costs (e.g., cash outlays to support the functioning of the project), and the economic costs (e.g., staff time not paid for by the project, MNHPs time and transport). An “ingredients approach” was utilized for the quantification and valuation of inputs (Drummond et al. 2015). These costs are specific to the Sierra Leone context. We did not include valuations of societal costs, such as the opportunity costs of investing in alternative programs (e.g., the benefits measured in life years saved of investing in the next best alternative health program), the time and travel costs of patients and family, and valuations of societal benefits (e.g., the value of productivity gains from mortality averted and the benefits to patients and family outside of the health gains). This choice was made largely for reasons related to the difficulty of identifying and quantifying such costs and benefits. Capital costs (e.g., equipment and vehicles) were assigned resale prices. Each cost item was apportioned to the two intervention arms (HP and HP+) in proportion to the contribution of each arm to the cost or based on estimated time committed to the different arms. An exchange rate of US$ 1 = 4050 Le (the local currency) was used. The rate ranged from 3750 to 4350 during the project period.

Cost estimates were derived from the project’s financial system, with the exception of opportunity costs, whose estimates were derived as follows:

Maternal Child Health (MCH) Aide Opportunity Costs: One MCH Aide at each of the 18 target PHUs was estimated to spend 3 days per month on supervision visits, meetings and handling of referral forms at PHUs. The salary used was US$ 55 per month, as reported in another study (Frontline Health Workers Coalition 2015).

MNHP Opportunity Costs: Project reports suggest each MNHP spent about 9 days per month conducting home visits, accompanying women to PHUs for delivery, and participating in monthly meetings. According to the World Bank, Sierra Leone’s GNI per capita in current US$ was $420 in 2010 and $490 in 2016, for an average value of $478.30 during the project period (interpreted as the annual earning for an average Sierra Leonean worker). Estimated MNHP earnings were discounted by 40% to account for the fact that MNHPs are unskilled workers. Each MNHP received a non-cash incentive valuing 5000 Le during the monthly meetings.

MNHP Transportation Not Covered by the Project: Project monitoring data shows that each MNHP made an average of 10 referrals per month, a third of which were for delivery. Generally, women were accompanied by MNHPs for delivery. The cost incurred by MNHPs to accompany women to the health facility was zero when the PHU was within walking distance and ranged from 500 to 5000 Le (one way) when another form of transportation was required. An estimated median value of 1500 Le was used for this evaluation.

### Analytical methods

#### Evaluating effectiveness

Difference-in-differences (DID) regression models (Heckman 2005; Bertrand et al. 2004) were used to quantify the impact of the intervention on the target outcomes, according to following Eq. (1):

1 $$ {\text{Ln}}\left( {\frac{{P_{ijt} }}{{1 - P_{ijt} }}} \right) = \beta_{0} + \beta_{1} A_{j} + \beta_{2} P_{t} + \beta_{3} A_{j} P_{t} + X\emptyset + \varepsilon_{ijt} . $$

where *P*_*ijt*_ = Pr (*Y*_*ijt*_ = 1); *Y*_*ijt*_ is an outcome for a woman *i* (*i* = 1, 2, … *Nj*), from enumeration area (EA) *j* (*j* = 1, 2, … *Mt*) at time *t* (*t* = 0, 1). *A*_*j*_ represents the study arm (indexed on the EAs), taking the values 1, 2 and 3 for the comparison arm, HP only arm and HP+ arm, respectively. *P*_*t*_ is a dummy variable coded as 0 for baseline and 1 for endline; *X* is a vector of individual level covariates (household wealth and respondent’s education, age and religion in this case); *β*_0_, *β*_1_, *β*_2_, *β*_3_ and $\varnothing$ are the corresponding regression coefficients; and *ε*_*ijt*_ is the error term clustered by EA. The DID estimate of interest is the coefficient *β*_3_ of the interaction between the variables *A*_*j*_ and *P*_*t*_.

Four versions of the above model were run, comparing each of the two intervention arms with the comparison group, comparing the two intervention groups with each another, and comparing the combined intervention groups with the comparison arm. STATA 14 software was used for the analysis.

#### Assessing cost-effectiveness

In the cost-effectiveness analysis, we use standard guidelines for economic evaluations of health interventions (Drummond et al. 2015), including the use of the Consolidated Health Economic Evaluation Reporting Standards (CHEERS) checklist (Husereau et al 2013). To estimate the number of maternal and child lives saved from improvements in the coverage of the outcomes of interest, we use the Lives Saved Tool (LiST) of Spectrum (version 5.47). LiST is a modeling software developed to estimate the impact of scaling up health and nutrition interventions for maternal, newborn and child health (Walker et al. 2013; Winfrey et al. 2011). The LiST model requires baseline and endline prevalence estimates of the outcomes and makes use of the built-in country-specific demographic data (Sierra Leone in this case). These indicators were generated for each arm using predicted probabilities from the logistic regression model in Eq. (1) above. The LiST model for each study arm was run, and national-level lives saved according to the population of the study arm were prorated and generated. The lives saved were further converted into discounted life years saved, making a number of assumptions including a 5% discount rate (to account for future benefits being weighed differently than present benefits), an average age of child death of 1 month, and a life expectancy of 50 years. As a result, each death averted was associated with approximately 49.9 years of life gained, which discounted at a 5% rate, amounted to 19.26 discounted years of life gained for every death averted.

The measure of cost-effectiveness, the incremental cost-effectiveness ratio (ICER), is defined as (CI-CC)/(MI-MC), where CI and CC are the total costs related to the intervention and comparison groups, respectively; and MI-MC are the life years saved in the intervention and comparison groups, respectively. Because the lives saved are incremental, CC = 0. ICER thus represents the average incremental cost associated with one additional life year saved. Our interpretation of cost-effectiveness is based on the World Health Organization (WHO)’s recommendations (WHO 2014). Interventions are classified as highly cost-effective if ICER is less than the country’s GDP per capita (US$638.3 for Sierra Leone), and cost-effective if ICER is less than three times the country’s GDP per capita (US$1915 for Sierra Leone).

## Results

### Effectiveness

The adjusted prevalence of the target outcomes at baseline, and the change from baseline to endline, is shown in Table 2. The coverage of four or more ANC visits during pregnancy increased markedly in the HP+ arm and was already near universal at baseline in the two other arms. While health facility delivery went up in all three arms, PNC for mothers dropped in the comparison communities and increased in the HP+ arm, and PNC for newborns declined in the comparison arm. Initiation of breastfeeding within 1 h of delivery improved significantly in both intervention arms.

**Table 2:** Cost of the Essential Newborn Care Corps project (Essential Newborn Care Corps Evaluation, Sierra Leone, 2014–2016)

Cost item	Amount (US$)	
	Total	%
Personnel, staff time and opportunity cost	$619,521	47.6
Personnel—concern and HPA Sierra Leone (SL)	$580,467	44.6
Opportunity cost—MCH Aides at PHUs^a^	$2835	0.2
Opportunity cost—MNHPs^a^	$36,218	2.8
Gross opportunity cost^a^	$41,358	3.2
Non-cash incentive^a^	− $5140	− 0.4
Travels and transportation	$207,897	16.0
Concern and HPA SL; MNHPs; MCH Aides and DHMT	$197,618	15.2
Non-covered by the project—MNHPs^a^	$10,279	0.8
Training and meetings	$123,755	9.5
Supplies and communications	$89,895	6.9
Equipment and rentals	150,125	11.5
Other direct costs and indirect costs	$111,302	8.5
Total project cost	$1,302,496	100.0
Estimates of break down per arm		
Health Promotion (HP) only arm	$582,151	44.7
Health Promotion plus Business (HP+) arm	$720,345	55.3

*HPA* Health Poverty Action, *PHU* Peripheral Health Unit, *DHMT* District Health Management Team, *MCH* Maternal and Child Health, *MNHP* Maternal and Newborn Health Promoter

^a^Not derived from the project’s financial records

Table 3 presents the impact evaluation estimates. The comparison of each intervention arm with the comparison group shows a statistically significant effect of the project in both intervention arms on breastfeeding initiation, PNC for mothers and PNC for newborns. The HP+ intervention had a statistically significant effect on health facility delivery and four or more ANC visits during pregnancy. The project does not seem to have impacted ANC initiation within the first trimester of pregnancy, despite the lower baseline values.

**Table 3:** Adjusted baseline indicators and change from baseline to endline (Essential Newborn Care Corps Evaluation, Sierra Leone, 2014–2016)

	Comparison arm		Health Promotion (HP) only arm		Health Promotion plus Business (HP+) arm		Combined intervention arms	
	Baseline	Change^a^	Baseline	Change	Baseline	Change	Baseline	Change
Primary outcomes								
ANC^b^ in 1st trimester	50.1	− 0.3	48.4	4.9	44.0	8.6	46.2	6.8
ANC 4 + visits	92.7	1.8	92.3	2.3	79.6	15.9***	85.9	9.3**
Health facility delivery	85.9	9.5***	72.5	11.4*	63.1	22.4***	68.0	16.7***
Postnatal care, mothers	85.8	− 11.8**	82.2	6.8	74.4	11.2*	78.3	8.9
Postnatal care, newborns	94.8	− 17.9***	92.1	− 1.0	90.4	− 3.5	91.3	− 2.4
Breastfeeding < 1 h	81.4	0.7	63.2	20.4**	70.4	25.3**	66.5	23.5**
Secondary outcomes^c^								
Tetanus toxoid vaccination	99.4	0.6	99.4	− 0.1	95.0	1.5	97.2	0.9
Iron supplementation	98.7	0.7	93.1	− 2.2**	86.5	11.2***	89.9	4.8
Malaria preventive treatment	89.5	2.7	83.1	8.0	85.0	5.6	84.2	6.5
Number of women	166	NA	180	NA	164	NA	344	NA

Among women who had a live birth in the 2 years preceding the survey

*p < 0.10; **p < 0.05; ***p < 0.01

^a^Percentage points change from baseline to endline

^b^Antenatal care

^c^Outcomes included for use in the lives saved estimates

Comparing the two intervention arms with one another, it appears the HP+ intervention had a significant additional effect over health promotion only on health facility delivery and four or more ANC visits. For PNC for mothers and breastfeeding, the difference-in-difference estimates also emerged in the same direction, but failed to reach statistical significance.

### Project cost

The cost associated with the HP arm and HP+ arm was estimated at US$ 582,151 and US$ 720,345, as shown in Table 4. Personnel costs accounted for 47.6% of the total costs. Limiting costs to only those incurred in-country, staff time at headquarters was excluded. Travel and transportation costs represented 16.0%. Likewise, travel costs for a learning meeting in Ghana were excluded. The remaining costs included equipment and rentals (11.5%), training and meetings (9.5%), other direct and indirect costs (8.5%), and supplies and communications (6.9%).

**Table 4:** Difference in differences estimates of the impact of the intervention on target outcomes (Essential Newborn Care Corps Evaluation, Sierra Leone, 2014–2016)

	HP only^a^ intervention versus comparison	HP+^b^ intervention versus comparison	Combined intervention arms versus comparison	HP+ intervention versus HP only intervention
Primary outcomes				
ANC^c^ in 1st trimester	5.2	8.9	7.1	3.7
ANC 4 + visits	0.5	14.1*	7.5	13.6**
Health facility delivery	1.9	12.9*	7.3	11.0*
Postpartum care, mothers	18.5**	22.9**	20.6**	4.4
Postnatal care, newborns	16.9***	14.4**	15.5**	− 2.6
Breastfeeding < 1 h	19.6*	24.6***	22.8**	4.9
Secondary outcomes^d^				
Tetanus toxoid vaccination	− 0.7	0.9	0.3	1.6
Iron supplementation	− 3.0	10.5	4.0	13.5*
Malaria preventive treatment	5.3	2.9	3.7	− 2.4

*p < 0.10; **p < 0.05; ***p < 0.001

^a^Health Promotion only arm

^b^Health Promotion plus Business arm

^c^Antenatal care

^d^Outcomes included for use in the lives saved estimates

### Cost-effectiveness

As shown in Table 5, the HP only intervention was associated with 141.0 life years saved relative to the comparison area, while the HP+ strategy generated 468.2 life years saved, despite the target population of the latter being about 20% smaller (46,355 compared with 57,040). The more than three times higher life years saved in the HP+ arm is attributable mainly to much larger increases relative to the control in health facility delivery and four or more ANC visits (DID estimates of + 11.0 and + 13.6, respectively, as shown in Table 4), and to a lesser degree, in iron supplementation during pregnancy (DID estimates of + 13.5).

**Table 5:** Lives saved from, and cost-effectiveness analysis of the Essential Newborn Care Corps intervention (Essential Newborn Care Corps Evaluation, Sierra Leone, 2014–2016)

	HP only^a^ intervention		HP+^b^ intervention		Combined HP only and HP+ intervention	
	Control arm	HP only arm	Control arm	HP+ arm	Control arm	HP and HP+ arms
Project areas population	57,040	57,040	46,355	46,355	103,395	103,395
Life years saved^c^	526.0	667.0	427.5	895.5	953.5	1622.7
Incremental life years saved		141.0		468.1		669.3
Project costs		$582,151		$720,345		$1,302,496
Per capita project cost		$10.2		$15.5		$12.6
Incremental cost-effectiveness ratio (ICER)^d^		$4130		$1539		$1946
ICER/GDP^e^ per capita		6.47		2.41		3.05

^a^Health Promotion only intervention arm

^b^Health Promotion plus Business intervention arm

^c^Lives saved estimates from the LiST software, multiplied by 19.26

^d^i.e., cost per one life-year saved

^e^Sierra Leone gross domestic product—per capita GDP is estimated at US$ 638.3 (Source: World Bank)

These life years saved from improved outcomes yielded an incremental cost-effectiveness ratio of US$4130 per life year saved in the HP only arm, and US$1539 per life year saved in the HP+ arm, which represent 6.47 times and 2.41 times the country’s gross domestic product per capita, respectively. Therefore, only the HP+ intervention is considered to be cost-effective, according to WHO standards.

## Discussion

This study sheds some light on the discourse by contemporary health systems researchers regarding the appropriate roles TBAs should play in the provision of perinatal care. This is particularly relevant to poor settings where there are no short-term alternatives to these trusted health resources who are highly valued members of their communities and are often the sole source of information and advice regarding pregnancy and childbirth.

The results show that the ENCC strategy was effective in increasing postnatal checkups for mothers and newborns in both intervention arms. The postnatal period is a critical period in the lives of mothers and newborns, with most maternal and infant deaths occurring during this window. Furthermore, early postnatal care is critical to promote healthy practices such as breastfeeding and infant vaccination. Yet PNC has not always received the attention, it deserves in MNCH programs (Langlois et al. 2015; Sines et al. 2017). Breastfeeding initiation within 1 h after birth also improved in the two intervention groups as a result of the ENCC project. Evidence suggests that increased risk of neonatal mortality is associated with increased delay in initiation of breastfeeding (Edmond et al. 2006).

The effect of the project on health facility delivery and the frequency of ANC visits during pregnancy was recorded in the HP+ group only, further suggesting that the business model had an added effect over and above health promotion on these two outcomes. The investigation of the reasons for this added advantage is beyond the scope of this paper. Monitoring data and other data sources show that both intervention arms had 100% retention of the rebranded health promoters and comparable levels of their expressed motivation. A comparable volume of referrals across time was also recorded. It may be argued, however, that with the prospects of income from the business, health promoters in the HP+ arm may have deliberately or unconsciously worked harder to motivate their clients during home visits (Sines et al. 2007).

Our findings on the effectiveness of the intervention should be interpreted in light of the following limitations. The baseline values of the target indicators were relatively high, making it hard for the project to effect any change. Further, on most indicators, these baseline values appeared higher in the comparison group, and lower in the HP+ arm, suggesting our effectiveness may be overestimated. The project was interrupted for about 6 months at the onset of the Ebola disease in August 2014, leaving a total implementation period of about 21 months, which may be short for any behavior change intervention to take root and generate impact. Finally, the sample size was relatively small (510 women at baseline and 753 women at endline).

Our study shows that the HP+ strategy of the ENCC model is cost-effective. While the HP+ strategy was about 55% more expensive per head than the HP only intervention (US$16.3 vs US$10.4 per head), the former generated far more life years saved, relative to the control, than the latter (10.1 vs 2.5 life years saved per 1000 population). This resulted from greater effectiveness drawn from improvements in health facility delivery and the frequency of ANC visits. A review of cost-effective strategies for MNCH concludes that while community-based packages have been and remain important, sustained efforts should be made to scale up the coverage of skilled birth attendance (Winfrey et al. 2011). Ultimately, the cost-effectiveness ratio of the HP+ intervention was nearly 60% lower than that of the HP only strategy (US$1539 vs US$4130 per life year saved).

As with any cost-effectiveness analysis, a few limitations that need to be considered. First, our cost analysis covers health sector costs only and does not include any private household costs, such as the opportunity costs. Likewise, it does not include value of productivity gains from mortality averted and the benefits to patients and family outside of the health gains. As Mangham-Jefferies et al. noted, cost-effectiveness analysis that does not take into account household costs, the values of donated goods, and related supply-side or demand-side costs, will underestimate the resources required to reduce maternal and neonatal mortality (Drummond et al. 2015). Second, had the cost-effectiveness analysis been included in the initial evaluation design, data on exclusive breastfeeding and family planning would have been collected and used to strengthen the robustness of the lives saved estimates. The ENCC project was not designed to be cost-effective; as the interim findings began to emerge, showing that the intervention had a potential to be adopted by the government, in-country stakeholders expressed interest in cost-effectiveness assessment.

### Conclusion

This study has shown that it is possible in resource-constrained settings to train and rebrand illiterate TBAs to promote perinatal care and institution-based delivery, thus serving as the linkage between their communities and the health system. Further, the strategy is effective in improving maternal and newborn care outcomes, and coupled with a small business model to incentivize TBAs, is cost-effective. As governments and development partners strive to improve MNCH in a context of scarce resources, it is critical to identify and scale down less cost-effective interventions, and reallocate resources to cost-effective options.

## References

[CR1] Adam T, Lim SS, Mehta S, Bhutta ZA (2005). Cost effectiveness analysis of strategies for maternal and neonatal health in developing countries. BMJ.

[CR2] Bertrand M, Duflo E, Mullainathan S (2004). How much should we trust differences-in-differences estimates?. Q J Econ.

[CR3] Campbell OM, Graham WJ (2006). Strategies for reducing maternal mortality: getting on with what works. Lancet.

[CR4] Curry LA, Byam P, Linnander E, Andersson KM (2013). Evaluation of the Ethiopian millennium rural initiative: impact on mortality and cost-effectiveness. PLoS ONE.

[CR5] Dorwie FM, Pacquiao DF (2014). Practices of traditional birth attendants in Sierra Leone and perceptions by mothers and health professionals familiar with their care. J Transcult Nurs.

[CR6] Drummond MF, Sculpher MJ, Claxton K, Stoddart GL, Torrance GW (2015). Methods for the economic evaluation of health care programmes.

[CR7] Edmond KM, Zandoh C, Quigley MA, Amenga-Etego S, Owusu-Agyei S, Kirkwood BR (2006). Delayed breastfeeding initiation increases risk of neonatal mortality. Pediatrics.

[CR8] Frontline Health Workers Coalition (2015) Cost of scaling up the health workforce in Liberia, Sierra Leone, and Guinea Amid the Ebola Epidemic. Frontline health workers coalition analysis

[CR9] Garces A, McClure EM, Chomba E, Patel A (2012). Home birth attendants in low income countries: who are they and what do they do?. BMC Pregnancy Childbirth.

[CR10] Government of Sierra Leone (2009) Free healthcare services for pregnant and lactating women and young children in Sierra Leone. In: Sierra Leone conference: stability, opportunity, growth

[CR11] Heckman J (2005). The Scientific model of causality. Sociol Methodol.

[CR12] Husereau D, Drummond M, Petrou S, Greenberg D, Augustovski F, Briggs AH, Mauskopf J, Loder E (2013). Consolidated health economic evaluation reporting standards (CHEERS) statement. Br Med J.

[CR13] Kayombo E (2013). Impact of training traditional birth attendants on maternal mortality and morbidity in sub-Saharan African countries. Tanzan J Health Res.

[CR14] Langlois EV, Miszkurka M, Zunzunegui MV, Ghaffar A, Ziegler D, Karp I (2015). Inequities in postnatal care in low- and middle-income countries: a systematic review and meta-analysis. Bull World Health Organ.

[CR15] Maitra C, Hodge A, Jimenez-Soto E (2016). A scoping review of cost benefit analysis in reproductive, maternal, newborn and child health: what we know and what are the gaps?. Health Policy Plan.

[CR16] Matendo R, Engmann C, Ditekemena J, Gado J (2011). Reduced perinatal mortality following enhanced training of birth attendants in the Democratic Republic of Congo: a time-dependent effect. BMC Med.

[CR17] Olufunke E, Akintujoye IA (2012). Perception and utilization of traditional birth attendants by pregnant women attending primary health care clinics in a rural Local Government Area in Ogun States, Nigeria. Int J Women’s Health.

[CR18] Oyerinde K, Harding Y, Amara P, Garbrah-Aidoo N, Kanu R, Oulare M (2013). A qualitative evaluation of the choice of traditional birth attendants for maternity care in 2008 Sierra Leone: implications for universal skilled attendance at delivery. Matern Child Health J.

[CR19] Sabin LL, Knapp AB, MacLeod WB, Phiri-Mazala G (2012). Costs and cost-effectiveness of training traditional birth attendants to reduce neonatal mortality in the Lufwanyama Neonatal Survival study (LUNESP). PLoS ONE.

[CR20] Shiferaw S, Spigt M, Godefrooij M, Melkamu Y, Tekie M (2013). Why do women prefer home births in Ethiopia?. BMC Pregnancy Childbirth.

[CR21] Sibley LM, Sipe TA, Koblinsky M (2004). Does traditional birth attendant training increase use of antenatal care? A review of the evidence. J Midwifery Women’s Health.

[CR22] Sines E, Syed U, Wall S, Worley H (2007). Postnatal care: a critical opportunity to save mothers and newborns.

[CR23] Singh D, Negin J, Otim M, Orach CG, Cumming R (2015). The effect of payment and incentives on motivation and focus of community health workers: five case studies from low- and middle-income countries. Human Resour Health.

[CR24] Statistics Sierra Leone and ICF International (2014). Sierra Leone demographic and health survey 2013.

[CR25] Treacy L, Sagbakken M (2015). Exploration of perceptions and decision-making processes related to childbirth in rural Sierra Leone. BMC Pregnancy Childbirth.

[CR26] Igme UN (2017). Levels & trends in child mortality: report 2017, estimates developed by the UN inter-agency group for child mortality estimation.

[CR27] Walker N, Tam Y, Friberg IK (2013). Overview of the lives saved tool (LiST). BMC Public Health.

[CR29] WHO (2014). Choosing interventions that are cost-effective.

[CR30] WHO (2015a) Recommendations on health promotion interventions for maternal and newborn health. WHO, Geneva. https://apps.who.int/iris/bitstream/10665/172427/1/9789241508742_report_eng.pdf 26180864

[CR31] WHO (2015b) Trends in maternal mortality: 1990 to 2015: estimates by WHO, UNICEF, UNFPA, World Bank Group and the United Nations Population Division. WHO, Geneva. https://apps.who.int/iris/bitstream/10665/194254/1/9789241565141_eng.pdf?ua=1

[CR28] WHO (2010) Working with individuals, families and communities to improve maternal and newborn health. Department of Making Pregnancy Safer, Geneva. https://apps.who.int/iris/bitstream/10665/84547/3/WHO_MPS_09.04_eng.pdf

[CR32] Wilson A, Gallos ID, Plana N, Lissauer D (2011). Effectiveness of strategies incorporating training and support of traditional birth attendants on perinatal and maternal mortality: meta-analysis. BMJ.

[CR33] Winfrey W, McKinnon R, Stover J (2011). Methods used in the lives saved tool (LiST). BMC Public Health.

